# Design and Mixing Analysis of a Passive Micromixer Based on Curly Baffles

**DOI:** 10.3390/mi14091795

**Published:** 2023-09-20

**Authors:** Makhsuda Juraeva, Dong-Jin Kang

**Affiliations:** School of Mechanical Engineering, Yeungnam University, 280 Daehak-Ro, Gyeongsan 38541, Gyeongbuk, Republic of Korea; mjuraeva@ynu.ac.kr

**Keywords:** signal-to-noise analysis, design parameter, degree of mixing (DOM), curly baffles, most influential design parameter, mixing mechanism

## Abstract

A novel passive micromixer based on curly baffles is proposed and optimized through the signal-to-noise analysis of various design parameters. The mixing performance of the proposed design was evaluated across a wide Reynolds number range, from 0.1 to 80. Through the analysis, the most influential parameter was identified, and its value was found to be constant regardless of the mixing mechanism. The optimized design, refined using the signal-to-noise analysis, demonstrated a significant enhancement of mixing performance, particularly in the low Reynolds number range (Re< 10). The design set obtained at the diffusion dominance range shows the highest degree of mixing (DOM) in the low Reynolds number range of Re< 10, while the design set optimized for the convection dominance range exhibited the least pressure drop across the entire Reynolds number spectrum (Re< 80). The present design approach proved to be a practical tool for identifying the most influential design parameter and achieving excellent mixing and pressure drop characteristics. The enhancement is mainly due to the curvature of the most influential design parameter.

## 1. Introduction

Micromixers play a crucial role in microfluidic systems, which find wide applications in biomedical diagnostics, chemical analysis, and drug delivery [1,2,3]. The micromixer is used in these systems to homogenize the sample reagents on microscale chips. The primary objectives of micromixers include the reduction of reagent consumption, achieving rapid mixing, and ensuring portability [3,4]. Fast and efficient mixing at microscale dimensions is essential to meet these objectives and enhance the overall performance of microfluidic systems.

Despite its importance, microfluidic mixing frequently encounters challenges in rapid and efficient processes due to limitations posed by molecular diffusion [2]. The microscale dimensions of micromixers and slow fluid velocity results in low Reynolds numbers, which further contribute to slow and inefficient mixing. As a result, there is a pressing need to develop more efficient micromixers to improve the overall performance of microfluidic systems. The progress of the microfluidic industry depends heavily on the development of efficient micromixers. While various technologies have been proposed to enhance microfluidic mixing, it still remains an active area of research [1,2,3,4].

Technologies for enhancing mixing in microfluidic systems can be broadly classified as active or passive. Active micromixers necessitate an external energy source to perturb fluid flow and induce the formation of vortices. This energy is typically supplied through acoustic fields [5], magnetic fields [6], electric current [7], thermal energy [8,9], or pressure pulsation [10,11]. While active micromixers can forcefully control the behavior of fluid, they also have some drawbacks such as high energy consumption, intricate driving equipment, low throughput, and difficulty in fabrication [5]. These factors limit the practical application of active micromixers in microfluidic systems, especially in portable and cost-effective systems [12].

Contrarily, passive micromixers utilize geometric configurations to induce chaotic fluid flow, eliminating the necessity for any moving components. This design approach results in simplified and cost-effective integration across a diverse spectrum of microfluidic systems. Researchers have explored various geometric arrangements and modifications to generate chaotic flow fields. These include designs like staggered herringbone [13], channel wall twisting [14], repeated surface groove and baffles [15,16], block in the junction [17], split-and-recombine (SAR) [18,19,20], Tesla structure [21,22], stacking of mixing units in the cross-flow direction [23,24], convergent–divergent micromixer [25], and submergence of planar structures [26,27]. However, it is important to note that most passive micromixers exhibit effective mixing within a limited Reynolds number range.

The demand for fast mixing times, on the order of milliseconds, in biological and chemical applications has led to the need for a micromixer that can operate effectively in a wide range of Reynolds numbers Re < 100 [28,29,30,31,32]. Micromixing in this range of Reynolds numbers is governed by two distinct mechanisms: molecular diffusion and chaotic convection. Consequently, micromixing can be classified into three mixing regimes based on the dominant mechanism: molecular domination, transition, and convection domination. Among these three regimes, the mixing in the transition regime is least efficient and corresponding Reynolds number is in the range of approximately 0.5 to 10. The existing micromixer deigns have limitations in their ability to enhance mixing in the transition regime. A novel design concept is required to overcome these limitations and achieve efficient mixing performance across a wide Reynolds number range.

To improve mixing in the transition regime, some researchers have delved into complex three-dimensional (3D) structures. For instance, Xia et al. [33] introduced a two-layer crossing channel to improve the mixing at Re = 0.2. Hossain et al. [34] designed a passive micromixer based on the concept of three-dimensional serpentine SAR microchannel using a series of OH-shaped segments, resulting in a mixing index of 0.884 for Re = 30. However, 3D micromixers are expensive and difficult to fabricate compared to planar micromixer designs. As a result, many researchers are currently focusing on modifying planar structures to generate 3D flow characteristics, circumventing the complexities associated with full-fledged 3D designs.

Among various planar structures, baffles have garnered substantial attention for their efficacy in enhancing mixing. For instance, Borgohain et al. [35] proposed the use of curved ribs to enhance mixing at low-to-medium Reynolds numbers (0.125 ≲Re≲64). Kang [16] strategically positioned rectangular baffles along the channel wall in a cyclic pattern to generate vortices both in the cross-flow and transverse directions. Tsai et al. [36] radially placed rectangular baffles in a curved microchannel, inducing vortices in multiple directions. Sotowa et al. [37] showed that indentations and baffles attached to the micromixer wall enhance mixing through secondary flow in deep micro-channel reactors. Raza et al. [38] improved the mixing performance of a SAR micromixer by embedding baffles immediately after each SAR unit, resulting in significant enhancement across the Reynolds number range from 0.1 to 80. Chung et al. [39] proposed the implementation of rectangular baffles with side gaps, leading to significant enhancement in mixing performance at both diffusion-dominant (Re < 0.1) and convection-dominant regimes (Re > 40), achieving over 90% of the mixing index. Chen et al. [40] placed baffles on both sides of a microchannel based on the Koch fractal principle to enhance micromixing. While these modifications using baffles have improved mixing performance at low and high Reynolds numbers, further improvement is needed especially in the transition regime of Reynolds numbers (0.2 ≲Re≲10) to develop efficient micromixers that can operate over a wide range of Reynolds numbers. In addition, most of the studies mentioned above have focused on rectangular baffles, and the use of curved baffles has not been extensively explored yet.

Recent research has highlighted the efficacy of a novel technique, submerging planar structures, to significantly enhance the mixing performance of 2D passive micromixers, particularly in the transition regime of mixing. This design approach comes with the advantage of reducing the required pressure drop. For instance, Makhsuda et al. [26] demonstrated that the submergence of planar mixing cells spurred the generation of secondary vortices in the transverse direction, resulting in a remarkable 182% improvement in the degree of mixing (DOM) and a 44% reduction in required pressure drop at Re = 10. Hsiao et al. [41] submerged winglet pairs in a microchannel, achieving noticeable improvement in DOM over a wide range of Reynolds numbers (0.125≤Re≤64). The submergence of planar structures constitutes a straightforward modification applicable to any planar micromixer, and can be readily fabricated through a microfabrication technique like Xurography [42]. Xurography utilizes thin, pressure-sensitive double-sided adhesive flexible films so that the submergence zone can be simply tailored using a cutter plotter. By assembling the tailored film with the planar structure, a passive micromixer can be modified accordingly. For example, Martínez-López et al. [43] demonstrated the application of Xurography in the fabrication of a passive micromixer.

When designing a passive micromixer, achieving optimal mixing performance entails the optimization of various geometric parameters. These optimization studies can be broadly categorized into two groups. One optimization approach is based on a single criterion, mostly mixing index. The other approach is the multi-object optimization, which deals with multiple object functions including the mixing index [44]. As most optimization approaches assume a continuous variation of the object functions, they require excessive computational or experimental cost. Consequently, the Taguchi design of experiment (DOE) method [45] is widely used to reduce not only the number of experiments required, but also analyze the sensitivity of design parameters [41,46,47]. Another issue in optimization is that the optimized geometry shows noticeable dependence on the flow condition or Reynolds number, where the optimization is carried out. For example, Rasouli et al. [48] optimized the geometry of a micromixer based on the curved channel and rectangular baffles in the three different regimes of mixing: diffusion dominance, transition, and convection dominance. Accordingly, the design of a passive micromixer intended to operate across a wide Reynolds number range needs a more pragmatic approach that takes into account both the geometric parameters and the flow conditions.

In this paper, we designed a novel passive micromixer, combining circular baffles with the submergence technique. The present micromixer comprises six mixing units, each consisting of two opposing half circles and three circular baffles inside. The geometric design parameters were evaluated based on their influence on the mixing performance via signal-to-noise (SN) analysis. Based on the results, the most influential design parameter across the three regime of diffusion, transient and chaotic mixing was first identified and used to optimize the present micromixer. The mixing performance of the optimized micromixer was assessed by computing the DOM at the outlet and the required pressure load between the inlets and outlet. All numerical simulations were conducted using ANSYS^®^ Fluent 2021 R2 [49].

## 2. Governing Equations and Computational Procedure

The governing equations are the fluid flow equations for three-dimensional laminar incompressible flows, along with a species transport equation to compute the evolution of mixing. Therefore, the following continuity and Navier–Stokes equations are used;

(1) $$\left(\vec{u}\cdot \nabla \right)\vec{u}=-\frac{1}{\rho }\nabla p+\nu \nabla^{2}\vec{u}$$

(2) $$\nabla \cdot \vec{u}=0$$

where $\vec{u}$, *p*, and *ν* are the velocity vector, pressure, and kinematic viscosity, respectively. The evolution of mixing is simulated by solving an advection–diffusion equation;

(3) $$\left(\vec{u}\cdot \nabla \right)\varphi =D\nabla^{2}\varphi$$

where *D* and *φ* are the mass diffusivity and mass fraction of fluid A, respectively.

The governing equations were solved by using a commercial software, ANSYS^®^ FLUENT 2021 R2 [49] based on the finite volume method. The convective terms in Equations (1) and (3) were discretized using the QUICK (quadratic upstream interpolation for convective kinematics) scheme, which is a third-order accurate interpolation scheme. The velocity distribution at the two inlets was assumed as uniform, and the outflow condition was used at the outlet. The no-slip boundary condition was specified along the all walls. The mass fraction of fluid A is specified to be *φ* = 1 at inlet 1 and *φ* = 0 at inlet 2, which means that fluid A is introduced at inlet 1 and fluid B (with mass fraction of 0) is introduced at inlet 2.

*DOM* and mixing energy cost (*MEC*) are used to evaluate the mixing performance of the present micromixer. *DOM* is defined as follows;

(4) $$DOM=1-\frac{1}{\xi }\sqrt{\sum_{i=1}^{n}\frac{{\left(\varphi_{i}-\xi \right)}^{2}}{n}},$$

where *φ_i_* and *n* are the mass fraction of fluid A in the *i*th cell and the total number of cells, respectively. *ξ* = 0.5 represents the complete mixing of two fluids. *MEC* is used to evaluate the effectiveness of present micromixer and is defined in the following form [50,51]:

(5) MEC=∆pρumean2DOM×100,

where $u_{mean}$ is the average velocity at the outlet, and ∆p is the pressure load between the inlet and the outlet.

The properties of the fluid flowing into both inlets, including density, diffusion coefficient, and viscosity, were assumed to be identical to those of water. They are *ρ* = 997 kg/m^3^, *D* = 1.0 × 10^−10^ m^2^ s^−1^, and *ν =* 0.97 × 10^−6^ m^2^ s^−1^, respectively. The Reynolds number is defined as $Re=\frac{\rho U_{mean}d_{h}}{\mu }$, where $\rho ,\;{\;U}_{mean},\;{\;d}_{h},\;\mathrm{and}\;\mu$ indicate the density, the mean velocity at the outlet, the hydraulic diameter of the outlet channel, and the absolute viscosity of the fluid, respectively. The corresponding Schmidt (Sc) number, which is the ratio of the kinetic viscosity and mass diffusivity of the fluid, is approximately 10^4^.

## 3. Validation of the Numerical Study

In the context of simulations involving high Schmidt (Sc) numbers, the issue of numerical diffusion can significantly compromise the accuracy of the simulated results. To enhance the quantitative rigor of numerical solutions, several strategies have emerged: the utilization of particle-based simulation methodologies, exemplified by the Monte Carlo method [52], or the reduction of cell Peclet number for grid-based methods. In grid-based methods, the cell Peclet number plays a pivotal role and is defined as $Pe_{c}=\frac{U_{cell}l_{cell}}{D}$, with $U_{cell}$ and $l_{cell}$ denoting the local flow velocity and cell size, respectively. A compelling recommendation, as put forth by Bayareh [53], suggests maintaining the cell Peclet number $Pe_{c}\leq 2$ to obtain numerical solution characterized by negligible numerical diffusion effects. However, these approaches, whether based on the Monte Carlo method or the criterion of a cell Peclet number of $Pe_{c}\leq 2,$ come with substantial computational costs, making their application impractical within the scope of studies like the present one. As a practical approach, most numerical studies prefer to conduct a grid independence test by comparing numerical solutions with the corresponding experimental data [26,34]. In lieu of resorting to these computationally intensive remedies, a pragmatic approach adopted by many numerical investigators is employed in this paper. This procedure entails comparing the obtained numerical solutions with corresponding experimental data [46], thereby allowing researchers to establish the reliability of the numerical solutions by examining convergence patterns across different grid sizes.

**Figure 1:**
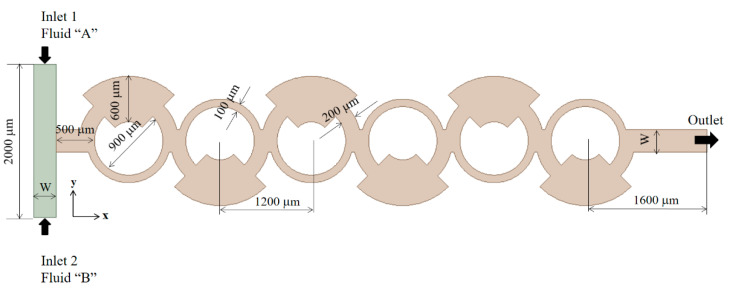
Diagram of the micromixer used by Xia et al. [54].

To validate the current numerical approach, a passive micromixer experimented by Xia et al. [54] was subjected to simulation. A schematic diagram of the micromixer is illustrated in Figure 1, where a rectangular cross section with dimensions of W = 300 μm width by 200 μm depth is maintained for both inlet channels. The micromixer incorporates six mixing units, each comprising a fan-shaped cavity, as depicted in Figure 1. To ensure consistency, the diffusion coefficient was assumed to align with the value reported by Xia et al. [54], measuring 1.2 × 10^−9^ m^2^/s. The simulations encompassed six different Reynolds numbers, specifically Re = 1, 10, 20, 40, 60, and 80. Following these simulations, a comprehensive comparison was conducted between the obtained results and the corresponding experimental data to demonstrate the accuracy and reliability of the current numerical approach.

**Figure 2:**
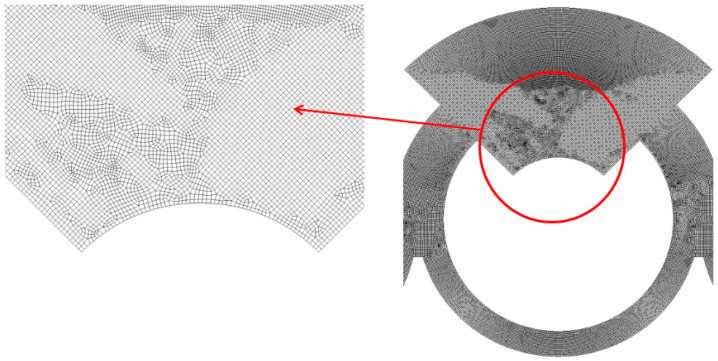
Grid within a mixing unit.

**Figure 3:**
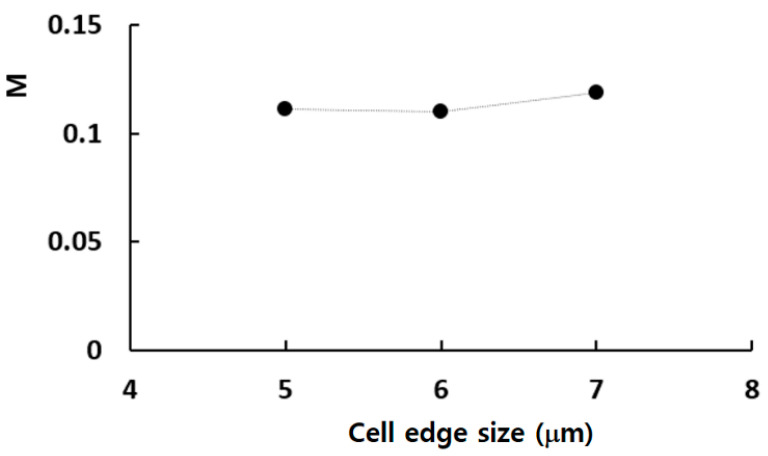
Grid independence of numerical solution.

The micromixer shown in Figure 1 was meshed using a sufficient number of cells. The edge size of each cell was determined through a set of preliminary simulations. To do this, the edge size was varied from 5 μm to 7 μm, corresponding to cell numbers ranging from 4.43 × 10^6^ to 11.5 × 10^6^. The simulations for this grid independence were conducted at a Reynolds number of Re = 10. Figure 2 presents an enlarged view of the grid within a mixing unit for a closer inspection. To enhance reliability in numerical results, hexahedral cells were predominantly employed in the present simulations. Figure 3 illustrates the grid independence of numerical solution. Here, *M* stands for the mixing index defined by Xia et al. [54] in the following way:

(6) $$M=1-\frac{\sigma_{D}}{\sigma_{D,o}},$$

and

(7) $$\sigma =\sqrt{\frac{1}{n}\sum_{i=1}^{n}{\left(\varphi_{i}-\varphi_{opm}\right)}^{2}},$$

where $\sigma_{D}$ is the standard deviation of φ, at any specific cross section normal to the flow, and $\sigma_{D,o}$ is the maximum standard deviation over the cross-section of the channel. *n* is the number of sampling points at any cross section and $\varphi_{i}$ is the mass fraction of fluid A at any sampling point *i*. $\varphi_{opm}$ is the optimal value of φ at any sampled cross section, which is set at 0.5 in this work. When the cell edge size is set to 6 μm, the relative error of numerical solution is reduced to 0.87% from 7.8% at 7 μm. Consequently, the simulation was carried out using an edge size of 6 μm, and the corresponding number of cells is 6.28 × 10^6^.

**Figure 4:**
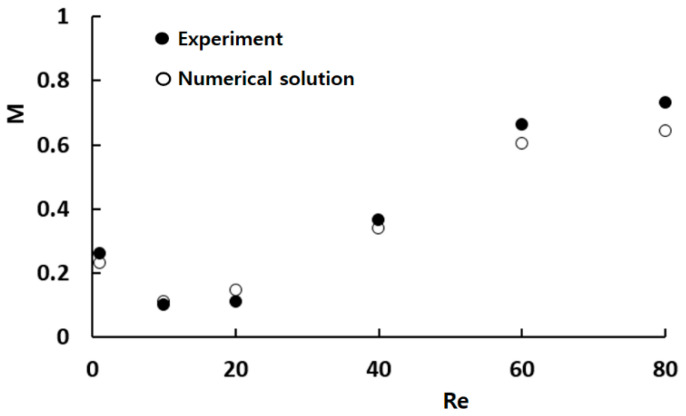
Comparison of numerical solutions with corresponding experimental data.

**Figure 5:**
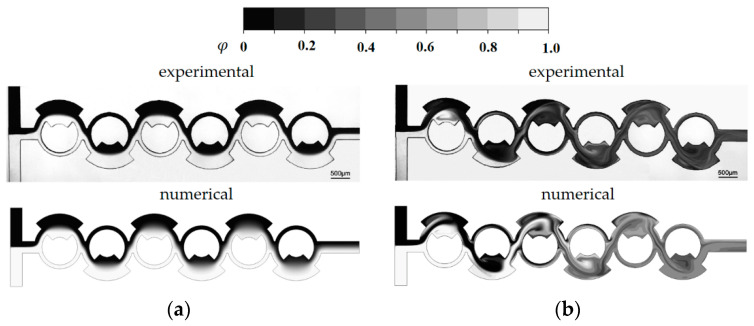
Comparison of numerical concentration contours with corresponding experimental images: (**a**) Re = 1 and (**b**) Re = 80.

Figure 4 presents a quantitative comparison between the numerical results and the corresponding experimental data by Xia et al. [54]. Despite some discrepancies between the numerical solution and experimental data in Figure 4, a similar trend was demonstrated with respect to the Reynolds number. The observed discrepancy could be ascribed to several factors, including numerical diffusion inherent in the simulation and experimental uncertainties inherent in experimental measurement. Figure 5 presents a comparison between the numerical concentration contours and the experimental confocal images at Reynolds numbers of Re = 1, and Re = 80. The simulated concentration contours show good agreement with the corresponding experimental images, irrespective of Reynolds number. Specifically, the mixing pattern due to the vortex motion within the mixing units is also predicted reliably at the Reynolds number of Re = 80.

## 4. Present Micromixer and Design Parameters

**Figure 6:**
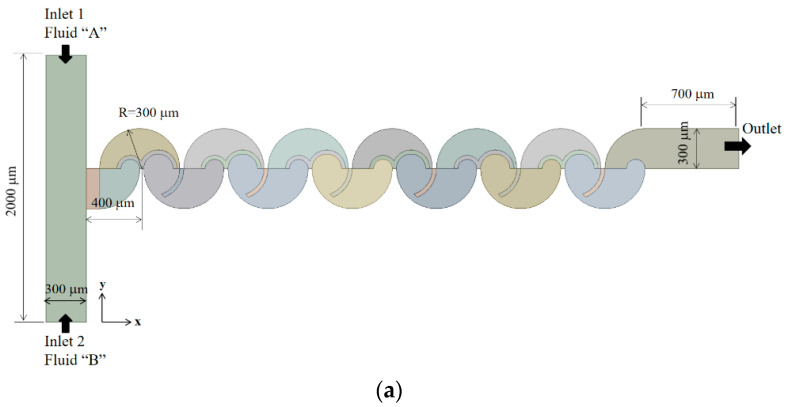

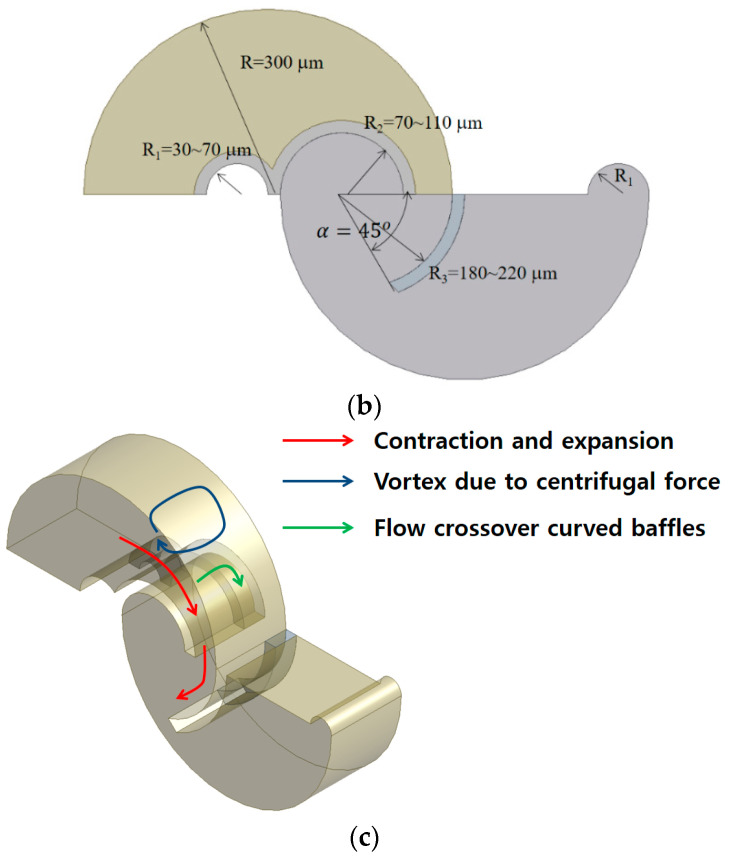
Schematic diagram of present micromixer: (**a**) front view, (**b**) detailed view of a mixing unit, and (**c**) distinct flow patterns in a mixing unit.

The present micromixer design comprises six mixing units, as depicted in Figure 6. Within each mixing unit, two mixing cells are integrated, each adopting a semi-circular configuration. These mixing cells house three circular baffles, with distinct radii denoted as R_1_, R_2_ and R_3_. It is noteworthy that the height of these circular baffles is shorter than the micromixer’s overall height, which measures 200 μm. As a result, the circular baffles are partially submerged in the z-direction.

The inlet and outlet branches of the micromixer feature a rectangular cross-section, with a width of 300 μm and a depth of 200 μm. Inlet 1 and inlet 2 both possess a length of 1000 μm, while the outlet branch spans 700 μm. Notably, the two inlets are positioned opposite to each other, and the primary mixing process transpires in the subsequent mixing units. The total length of the micromixer of six mixing units amounts to approximately 3.5 mm.

Figure 6b illustrates an in-depth view of a mixing unit. The three circular baffles within it are uniquely configured with different radii, denoted as R_1_, R_2_, and R_3_ to study their effects on the mixing performance. Specifically, R_1_ ranges from 30 μm to 70 μm, R_2_ spans from 70 μm to 110 μm, and R_3_ is varied from 180 μm to 220 μm. Through the utilization of the signal-to-noise analysis, the most suitable dimensions for these three baffles were identified and subsequently determined. The present mixing unit was specifically designed to facilitate three distinct flow patterns, as visually illustrated in Figure 6c. The first pattern involves flow through a contraction-and-expansion channel created by curved baffles. The second pattern entails a vortex flow induced by the centrifugal forces, and the third pattern encompasses flow crossover through submerged baffles. These diverse flow patterns are expected to enhance mixing performance across a wide spectrum of Reynolds numbers.

**Figure 7:**
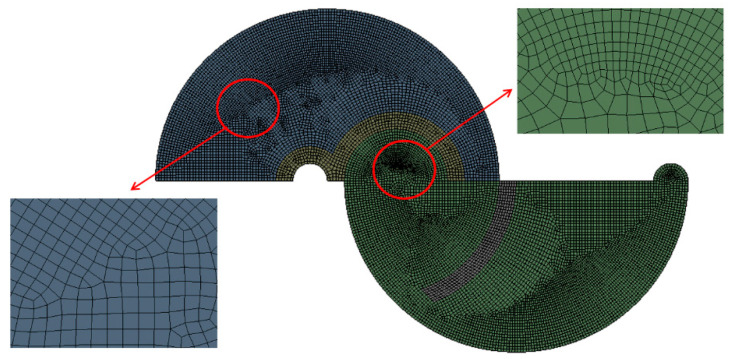
Example of grid in a mixing unit.

The computational domain shown in Figure 6 was meshed using a sufficient number of cells. To minimize any associated numerical diffusion, the size of each cell was determined through a set of preliminary simulations. The simulation was conducted at a Reynolds number of Re = 0.5. To this end, the edge size of each cell was varied from 4 μm to 6 μm, corresponding to cell counts ranging from 2.14 × 10^6^ to 10.4 × 10^6^. Figure 7 presents an enlarged view of the grid within a mixing unit for a closer inspection. Following the findings of Okuducu et al. [55], the type of cells used in simulations can significantly impact the accuracy of numerical solutions. To enhance reliability in numerical results, structured hexahedral cells were predominantly employed in the present simulations, as depicted in Figure 5. The utilization of prism cells was minimized, while tetrahedral cells were entirely avoided in this simulation.

The uncertainty of the numerical solution was assessed through the grid convergence index (*GCI*) [56,57], based on the simulation results. The *GCI* was computed using the following formula:

(8) $$GCI=F_{s}\frac{\left|\varepsilon \right|}{r^{p}-1},$$

where *F_s_*, *r*, and *p* represent the safety factor of the method, grid refinement ratio, and the order of accuracy of the numerical method, respectively. ε is determined by the equation:

(9) $$\varepsilon =\frac{f_{coarse}-f_{fine}}{f_{fine}},$$

where *f_coarse_* and *f_fine_* are the numerical solutions obtained with a coarse grid and fine grid, respectively. In this study, *F_s_* was set at 1.25, following the recommendation of Roache [56]. The edge sizes considered were 4 μm, 5 μm, and 6 μm, resulting in corresponding cell counts of 2.14 × 10^6^, 5.2 × 10^6^, and 10.4 × 10^6^, respectively. After evaluating the GCI of the computed DOM for each edge size, it was found that the GCI is approximately 0.8% when using an edge size of 5 μm. Consequently, the edge size of 5 μm was chosen to mesh the computational domain due to its favorable GCI value, ensuring a suitable balance between accuracy and computational cost.

The geometric parameters for the present design were optimized through a signal-to-noise (SN) analysis. A total of four geometric parameters, namely R_1_, R_2_, R_3_, and N, were chosen for this analysis. Here N represents the number of mixing units equipped with three circular baffles inside, varying from 4 to 6. While increasing N typically enhances DOM, it also leads to a higher pressure drop in the micromixer. However, Natsuhara et al. [58] demonstrated that N should be determined to achieve the highest DOM for a given micromixer size. The four design factors were assumed to have three levels, as listed in Table 1. The value of R_2_ determines the blockage ratio between the upper and lower mixing units. As R_2_ varies within the range of 70 to 110 μm, the blockage ratio ranges from 12% to 38% of the total width when there are no baffles in place. This particular range was selected based on the findings from Usefian [25], who demonstrated that the highest mixing efficiency was achieved with a blockage ratio of 25%. To streamline the simulation process, the orthogonal array L_9_(3^4^) was selected in alignment with the Taguchi method [45]. This choice reduced the simulations from 81 to 9, effectively conducting the analysis. These preliminary simulations were executed across three different Reynolds numbers of 0.5, 5, and 20. Each of these Reynolds numbers represents a specific mixing regime: molecular diffusion dominant, transient and chaotic convection dominant. This comprehensive assessment across all possible mixing regimes enables the evaluation of design factor effects on the mixing performance over a wide range of Reynolds numbers.

**Figure 8:**
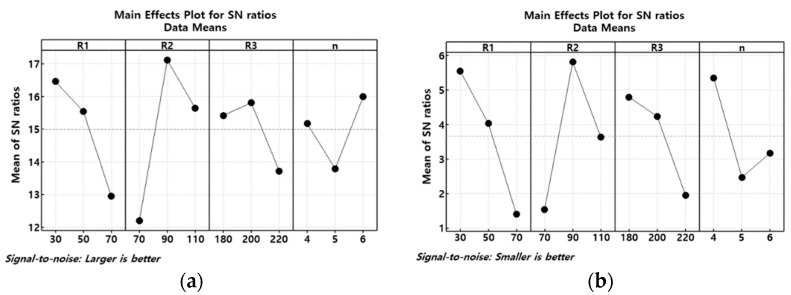
Influence of design parameters at Re = 0.5; (**a**) normalized DOM and (**b**) normalized MEC.

The effects of the design factors on the mixing performance were analyzed using the Taguchi method. In the context of signal-to noise (SN) analysis, both the DOM and MEC were normalized with respect to the micromixer without circular baffles. The principle of larger is better is employed to maximize the normalized DOM, while the principle of smaller is better is applied to minimize the normalized MEC. Figure 8 presents the analysis results in terms of the main effects for signal-to-noise ratio at Re = 0.5. The design factors exhibited distinct degrees of influence on the normalized DOM, with their impact arranged in the order of R_2_ > R_1_ > R_3_ > N. On the other hand, the rank of influence on the normalized MEC is arranged differently in the order of R_2_ > R_1_ > N > R_3_. Consequently, the most pronounced influence on the mixing performance of the present micromixer is R_2_, and an optimal value of 90 μm is identified. Other design parameters could be determined based on their impact, either at the highest value of normalized DOM or the lowest value of normalized MEC. In this regard, an optimized design with R_1_ = 30 μm, R_2_ = 90 μm, and R_3_ = 200 μm would maximize the normalized DOM within the given range of the four parameters (referred to as Case 1). In contrast, a design with R_1_ = 30 μm, R_2_ = 90 μm, and R_3_ = 180 μm minimizes the normalized MEC (referred to as Case 2). The number of mixing units having circular baffles, N, was compromised at 5.

We followed the same procedure for Re = 5 and 20 to investigate how the flow condition affects the geometric optimization for enhancing mixing in the micromixer under consideration. Table 2 provides a summary of the analysis results for the four geometric parameters. These parameters were determined based on the *main effects for signal-to-noise* ratio. At Re = 20, the convection plays a dominant role in the mixing process. Among the designs, the one with R_1_ = 50 μm maximizes the normalized DOM. This result suggests that the optimal value of geometric parameters depends on the dominant mechanism of mixing as well as the optimization object function. Based on the result summarized in Table 2, we have selected three optimized designs as listed in Table 3. An interesting finding is that the value of the most influential parameter does not show any dependence on the Reynolds number or the mixing mechanism. Table 3 demonstrates that the value of R_2_ remains constant at R_2_ = 90 μm for all the three cases. Further analysis was conducted to evaluate the mixing performance of these designs. Case 1 and Case 3 are optimized to maximize the normalized DOM. Specifically, Case 1 is chosen in the molecular dominance regime, while Case 3 is selected in the convection dominance regime. On the other hand, Case 2 represents the optimized design for minimizing the normalized MEC. Notably, the value of the most influential parameter R_2_ remains unaffected by the Reynolds number.

**Figure 9:**
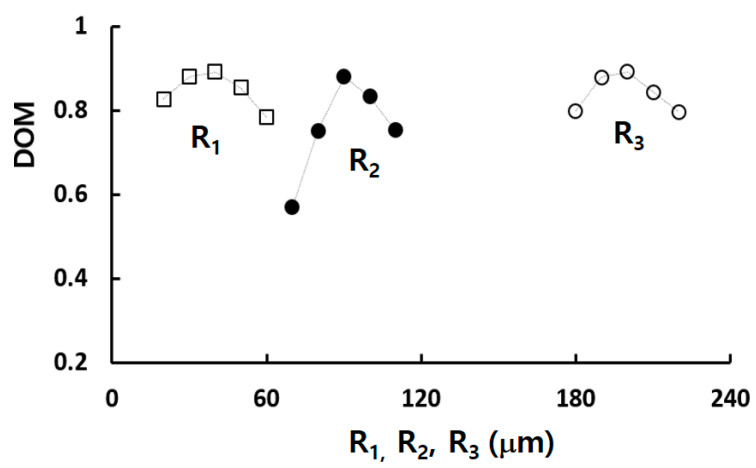
Effects of design geometric parameters on DOM at Re = 0.5: While one parameter varies within a specific range, the others are held constant at their respective base values. These base values for R_1_, R_2_, and R_3_ are 30, 90, and 200 μm, respectively.

Figure 9 demonstrates how the optimal values of R_1_, R_2_, and R_3_ affect the DOM at Re = 0.5. It confirms that the design values listed in Table 3 are properly optimized for the DOM. The figure also affirms that R_2_ is most influential parameter as the DOM shows the greatest dependence on R_2_. As seen in Figure 8, both R_2_ and R_3_ were selected at the peak value of the normalized DOM, resulting in the highest DOM at R_2_ = 90 μm and R_3_ = 200 μm, respectively. In contrast, R_1_ was chosen at the highest value of the normalized DOM in Figure 8, with the optimum value being R_1_ = 40 μm in Figure 9. Nevertheless, its effect on the DOM is relatively limited.

## 5. Mixing Performance of Optimized Designs

The mixing performance of three optimized designs was simulated over a wide Reynolds number range from 0.1 to 80. For these numerical simulations, a uniform velocity profile ranging from 0.25 mm/s to 0.2 m/s was imposed at the two inlets, thereby leading to volume flow rate ranging from 1.2 μL/min to 964.6 μL/min. The assessment of mixing performance entails the calculation of DOM at the outlet, along with the associated pressure drop.

**Figure 10:**
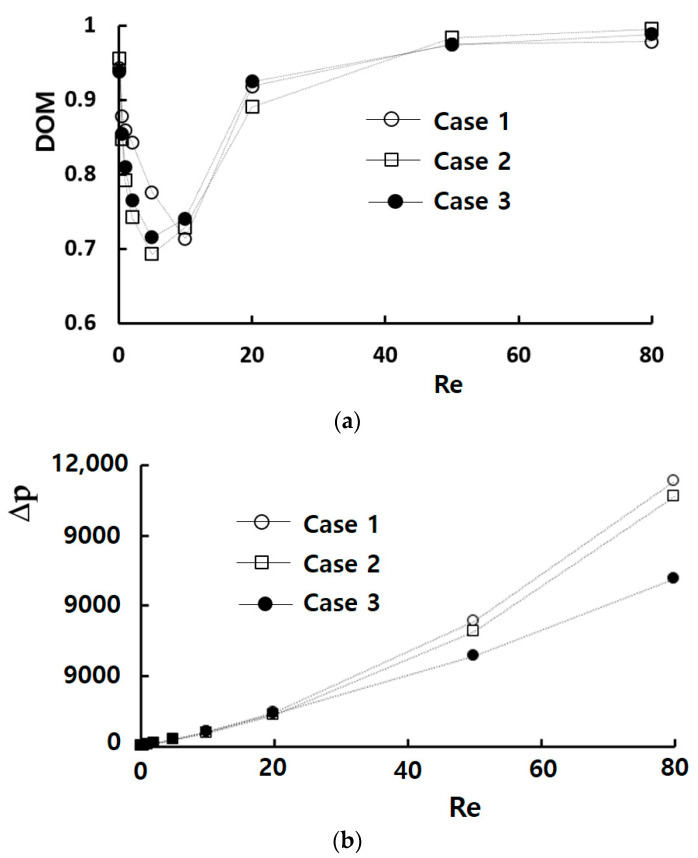
Comparison of three optimum designs: (**a**) DOM vs. Re, and (**b**) ∆p vs. Re.

Figure 10 compares the mixing performance of the three optimized designs. Case 1 shows the best DOM performance when the Reynolds number is approximately less than 10. For example, the DOM of Case 1 is increased 8% from that of Case 3 for Re = 5. On the other hand, Case 3 requires the minimum pressure drop when the Reynolds number is larger than 20. For instance, the require pressure drop of Case 3 is 58% reduced from that of Case 1 for Re = 80, even though Case 3 presents a slightly higher DOM. A noteworthy observation is that the DOM in the range of 0.1<Re < 10 is highly responsive to the optimized geometric parameter values. Consequently, Case 1 optimized for the molecular dominance mixing regime emerges as a preferable choice when designing a passive micromixer operating over a wide range of the Reynolds number.

**Figure 11:**
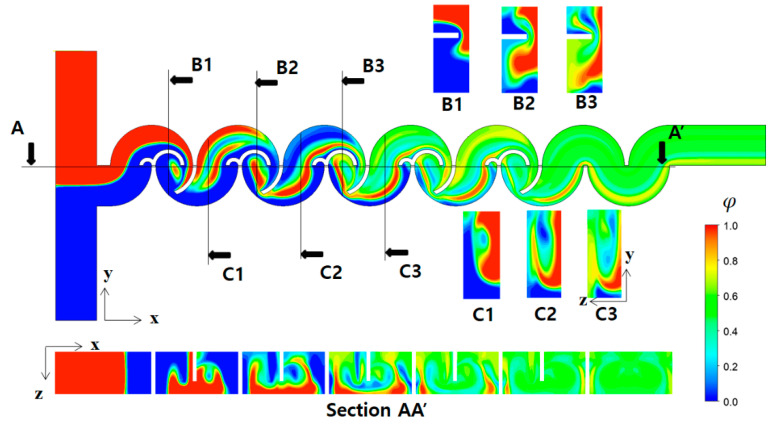
Evolution of mixing along the micromixer for R_1_ = 30 μm, R_2_ = 90 μm, and R_3_ = 200 μm at Re = 0.5.

**Figure 12:**
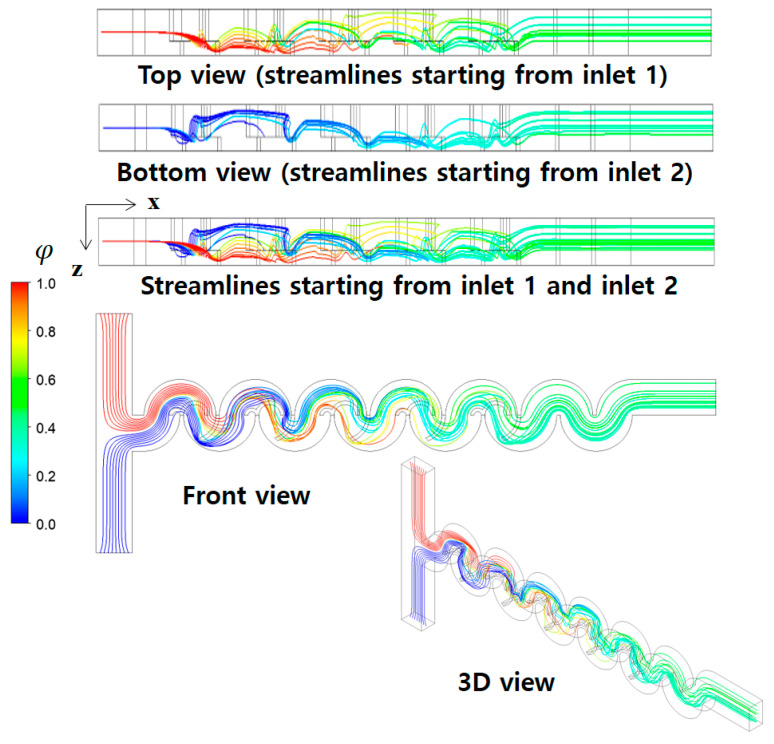
Development of streamlines starting from two inlets along the micromixer for R_1_ = 30 μm, R_2_ = 90 μm, and R_3_ = 200 μm at Re = 0.5.

Figure 11 illustrates the mixing process of Case 1 in terms of concentration contours at various cross sections at a Reynolds number of Re=0.5. The cross sections, B1, B2, B3, C1, C2, and C3, were obtained cutting the micromixer in the x-direction as indicated. Similarly, the plane AA′ is in the y-direction. The concentration contours on the xy plane unveil a vigorous mixing process taking place after the fluid traverses the second baffle within each mixing unit, leading to the envelopment of fluid “A” by fluid “B”. Upon closer examination of the concentration contours at the B1, B2, and B3 cross sections, it becomes evident that the submerged circular baffles operate as a contraction-and-expansion mixing device. This configuration effectively enhances mixing, as shown in previous research [59]. On the other hand, the concentration contours at C1, C2, and C3 cross sections demonstrate that a distinct mixing process occurring within the open space between the lower and upper mixing cells is achieved differently. This mixing enhancement is caused by the flow following the circular wall of the micromixer, with the walls facing opposite each other. In Figure 12, the mixing enhancement is further elucidated through streamlines. Upon entering the first mixing cell, streamlines originating from the both inlets initially travel separately in the z-direction. However, a convergence of streamlines is observed from the second mixing unit onwards, facilitated by the flow adhering to the circular walls. This unique flow pattern is an additional mechanism contributing to the mixing enhancement in the low Reynolds number regime (Re < 10). This phenomenon is visually apparent through the multiple fluid interfaces captured within the open space in Figure 11. In the open space connecting the lower mixing cell and the upper mixing cell, the concentration contours in Figure 11 show several elongated interfaces.

**Figure 13:**
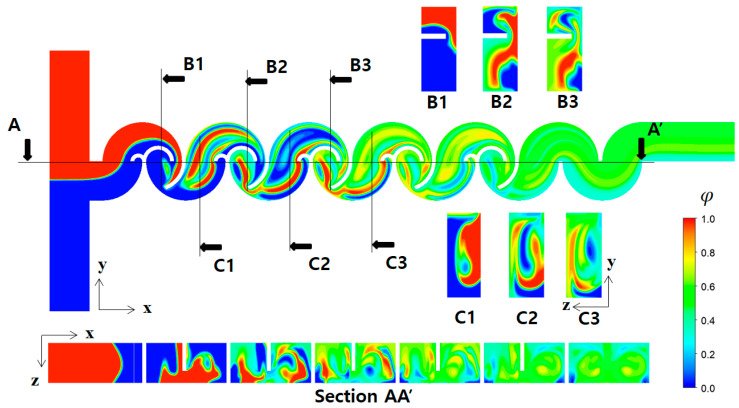
Evolution of mixing along the micromixer for R_1_ = 30 μm, R_2_ = 90 μm, and R_3_ = 200 μm at Re = 20.

**Figure 14:**
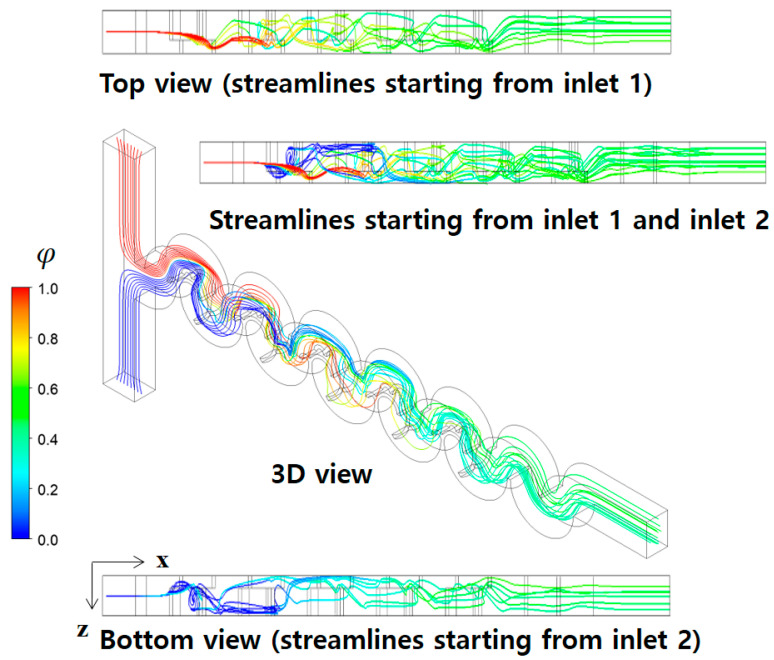
Development of streamlines starting from two inlets along the micromixer for R_1_ = 30 μm, R_2_ = 90 μm, and R_3_ = 200 μm at Re = 20.

**Figure 15:**
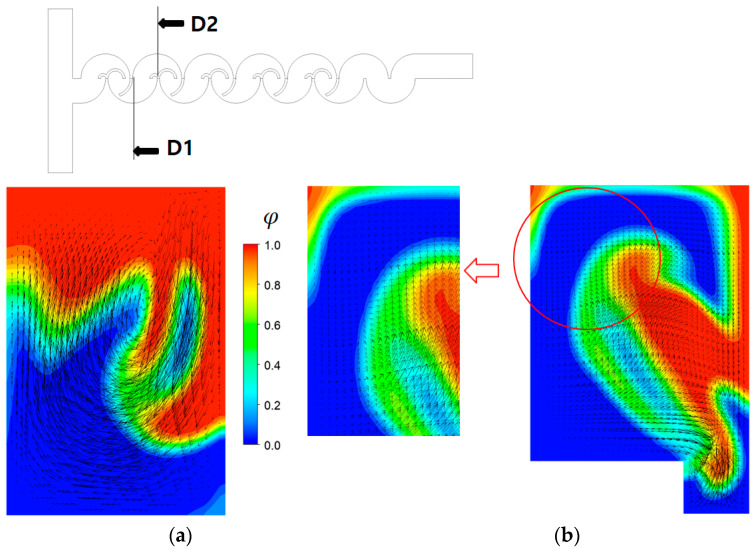
Vortex pattern and concentration contours at Re = 20 for R_1_ = 30 μm, R_2_ = 90 μm, and R_3_ = 200 μm at Re = 20: (**a**) at D1 and (**b**) at D2.

Figure 13 illustrates the concentration contours of fluid “A” at several cross sections at a Reynolds number of Re = 20. A comparison with Figure 11 reveals notably intensified mixing, particularly evident within the open space amid the lower and upper mixing cells. This is observed as more fluid interfaces in the figure. This enhanced mixing characteristic is explained in Figure 14 where the streamlines travel more widely in the z-direction, and cross each other as they travel downstream. In comparison with Figure 12, this vigorous flow pattern strongly suggests the formation of secondary vortices at the cross sections, as affirmed in Figure 15. It shows that a big vortex is formed at the cross sections of D1 and D2. Therefore, the mixing enhancement at Re = 20 is mainly caused by these secondary vortex, unlike the contraction-and-expansion mechanism due to the submergence of the baffles at Re = 0.5. Another difference is that the mixing process beneath the second baffle within each mixing unit is relatively weaker than that at Re = 0.5. This implies that the curly baffles are less effective to mixing at Reynolds numbers lager than Re = 20.

**Figure 16:**
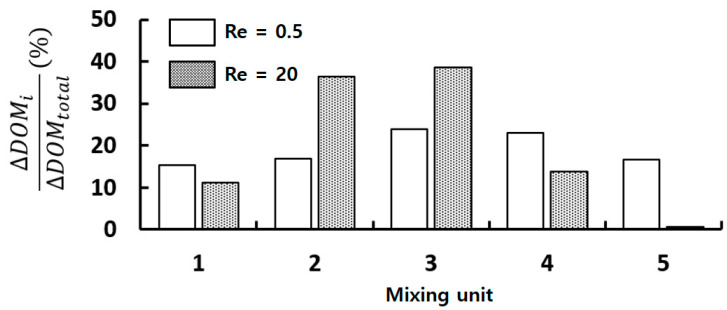
DOM increment at each mixing unit in percentage for R_1_ = 30 μm, R_2_ = 90 μm, and R_3_ = 200 μm.

Figure 16 provides a comprehensive comparison of the mixing contribution by each individual mixing unit within the micromixer, as a percentage of the overall DOM of the micromixer. At Re = 0.5, each mixing units contributes a similar amount across all five mixing units. This even contribution suggests that the set of three circular baffles effectively functions as a beneficial mixing device in the low Reynolds number regime (Re < 10). Conversely, at Re = 20, the contributions of the second and third mixing units are notably greater than those from the remaining mixing units. Intriguingly, the fifth mixing unit makes a negligible contribution to the overall mixing. This implies that the set number of baffles should be considered in the optimization of a micromixer operating across a wide Reynolds number range.

**Figure 17:**
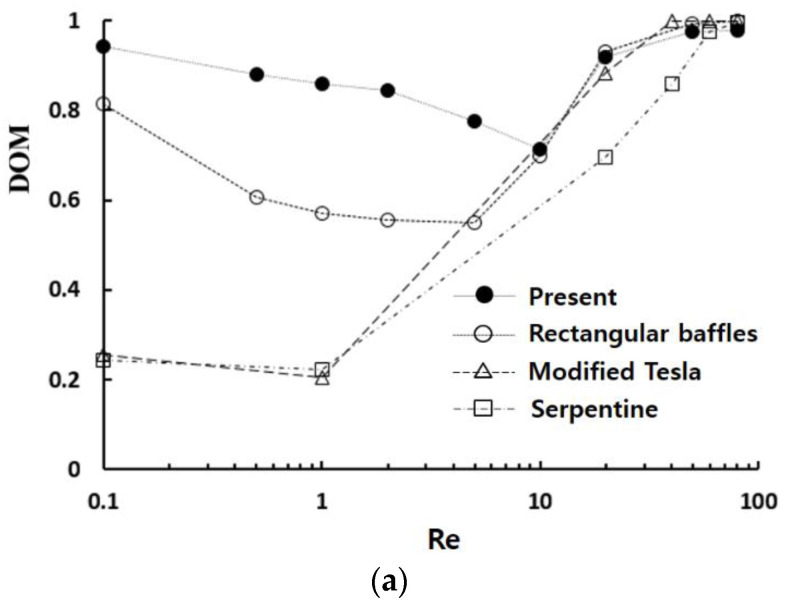

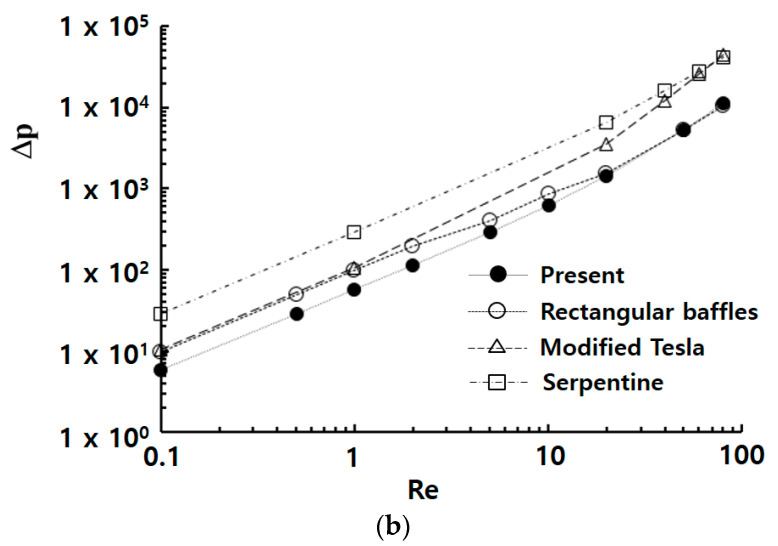
Comparison of the mixing performance of present micromixer with other passive micromixers: (**a**) DOM vs. Re, and (**b**) ∆p vs. Re.

Figure 17 presents a comparative assessment of mixing performance between the present micromixer and several conventional passive micromixers as a function of the Reynolds number: rectangular baffles [27], modified Tesla [21], and serpentine [60]. All four passive micromixers underwent simulations under identical boundary conditions and shared the same physical properties. The result demonstrates that the present micromixer based on baffles shows the best mixing performance micromixers in the lower Reynolds number regime (Re< 10), compared with other micromixers such as the modified Tesla and serpentine. For example, the DOM of present micromixer reaches approximately 0.86 at Re = 1, signifying a remarkable 51% increase over the micromixer employing rectangular baffles. This increase highlights the superior mixing performance of curly baffles when compared to rectangular baffles. Furthurmore, the present micromixer requires the lowest pressure drop among the four micromixers. Specifically, at Re = 1, the required pressure drop of the present micromixer is diminished by 40% compared to the micromixer using rectangular baffles. Interestingly, the present micromixer exhibits comparable mixing performance to the rectangular baffles and modified Tesla micromixers in the high Reynolds number regime (Re≥20). This observation suggests that the effects of baffle curvature becomes less significant in this Reynolds number range.

## 6. Conclusions

In this paper, a passive micromixer based on curly baffles is optimized using the signal-to-noise analysis. The micromixer comprises six distinct mixing units, each housing two mixing cells containing three circular baffles. Therefore, the radii of these three circular baffles represent the principal design parameters. Additionally, the mixing unit number having three circular baffles was also considered as a design parameter. To streamline the optimization process and minimize simulation efforts, we employed the orthogonal array L_9_(3^4^) in accordance with the Taguchi method. Computational simulations to evaluate the mixing performance of the present micromixer were conducted using the ANSYS^®^ Fluent 2021 R2.

The present design approach is based on a signal-to-noise analysis. It entails simulating the mixing performance at three distinct Reynolds numbers, which represents three different mixing regimes. Specifically, Re = 0.5 signifies the regime dominated by the molecular diffusion, Re = 20 corresponds to the convection dominance regime and Re = 5 characterizes the transitional mixing regime. This approach utilizes the DOM and the MEC as signal functions, with both normalized with respect to the micromixer without circular baffles. The larger is better principle was employed to maximize the normalized DOM, while the smaller is better principle was utilized to minimize the normalized MEC. Optimized values for the design parameters was determined by identifying the peak or highest mean value of the SN ratio.

Each design factor exhibits a distinct level of influence on the mixing performance. When optimizing the design factors at Re = 0.5, the level of influence for maximizing the normalized DOM was R_2_ > R_1_ > R_3_ > N. Conversely, for minimizing the normalized MEC, the rank of influence was R_2_ > R_1_ > N > R_3_. A noteworthy discovery is that the most influential design parameter governing mixing performance remains consistent and easily ascertained by the present design approach. Another significant finding is that the optimal value of the most influential design parameter is consistently determined, irrespective of the Reynolds number. The optimized combination of design parameters derived from the diffusion dominance range yields the highest DOM in the low Reynolds number range (Re< 10): R_1_ = 30 μm, R_2_ = 90 μm, and R_3_ = 200 μm. In contrast, the design set optimized for the convection dominance regime demonstrates minimal pressure drop across an extensive Reynolds number span (Re< 80): R_1_ = 50 μm, R_2_ = 90 μm, and R_3_ = 200 μm.

The present design approach has demonstrated its effectiveness in identifying the most influential parameter among a number of design parameters. In comparison to other typical passive micromixers, the present design yields a remarkable enhancement in DOM, especially in the low Reynolds number range of Re< 10, while simultaneously requiring the lowest pressure drop across the entire Reynolds number spectrum (Re< 80). This enhancement is primarily attributed to the curvature of the most influential design parameter, R_2_.

## Figures and Tables

**Table 1:** L_9_(3^4^) orthogonal array.

Case	Parameter			
	R_1_ (μm)	R_2_ (μm)	R_3_ (μm)	N
1	30	70	180	4
2	30	90	200	5
3	30	110	220	6
4	50	70	200	6
5	50	90	220	4
6	50	110	180	5
7	70	70	220	5
8	70	90	180	6
9	70	110	200	4

**Table 2:** Optimization of design parameters based on the signal-to-noise ratio.

	Re = 0.5		Re = 5		Re = 20	
	DOM	MEC	DOM	MEC	DOM	MEC
R_1_ (μm)	30	30	30	30	50	30
R_2_ (μm)	90	90	90	90	90	90
R_3_ (μm)	200	180	200	180	200	180
*n*	6	4	6	4	6	4

**Table 3:** Three designs optimized by the SN analysis.

	R_1_ (μm)	R_2_ (μm)	R_3_ (μm)
Case 1	30	90	200
Case 2	30	90	180
Case 3	50	90	200

## Data Availability

Not applicable.
